# Supplementary material for: Effects of electroporation on Acanthamoeba Polyphaga
Source: PLoS One. 2025 Feb 25;20(2):e0317409. doi: 10.1371/journal.pone.0317409 (PMC12072634; doi:10.1371/journal.pone.0317409)
Supplement: Attachment — Submitted filename: PPC-Report-176780.pdf [file pone.0317409.s001.pdf]

گزارش شباهت یابی

شناسه مقاله: ۱۷۶۷۸۰

۱۲:۳۵ - ۱۴۰۳/۳/۵

ppc.research.ac.ir

176780

WORD COUNT 5488

TIME SUBMITTED 25-MAY-2024 03:38PM

PAPER ID 109204741

PLOS ONE

Effects of electroporation on Acanthamoeba Polyphaga

--Manuscript Draft--

**Manuscript Number:**	PONE-D-24-12061
**Article Type:**	Research Article
**Full Title:**	Effects of electroporation on Acanthamoeba Polyphaga
**Short Title:**	Eletroporation on Acanthamoeba Polyphaga
**Corresponding Author:**	Palloma Santiago Prates Pessoa, B.Sc.<br>Universidade Federal de São Paulo: Universidade Federal de Sao Paulo<br>São Paulo, BRAZIL
**Keywords:**	Acanthamoeba, Acanthamoeba Keratitis, electroporation, therapie, eye.
**Abstract:**	Background: Species of the genus Acanthamoeba spp. are ubiquitous and can cause Acanthamoeba keratitis (AK), an opportunistic infection. Due to the growth in the number of AK cases, and therapies lasting an average of 6 months to an year, we are investigating the possibility of electrochemical treatment, which consists of destabilizing the membrane/wall of Acanthamoeba trophozoites and cysts to eliminate these forms more quickly. Therefore, we investigated whether electroporation could induce non-viable Acanthamoeba cysts and trophozoites in vitro.<br>Methods: Cysts and trophozoites of Acanthamoeba polyphaga (ATCC® 30461TM) were exposed in vitro to an electric field with intensities of 2000 volts and 2500 volts. The effect of electroporation on feasibility was assessed by counting in a Neubauer hemocytometer using Trypan blue 0,4% dye, and permeabilization was assessed by fluorescence microscopy using propidium iodide (PI) to identify permeabilized amoebas, associated with the nuclear dye 4',6-diamidino-2-phenylindole dihydrochloride (DAPI). The images were acquired on a Nikon Eclipse TI-U microscope using an excitation wavelength of 488/617 for PI and 340/488 nm for DAPI and analyzed using ImageJ.<br>Results: The results obtained demonstrated the loss of 40% of trophozoites electroporated at 2000V and 42% of those electroporated at 2500V, while for cysts the loss was respectively 13% and 16%. For permeabilization at 2000V, the rate was 55% for trophozoites and cysts ($p \leq 0.05$); while at 2500V it was 59% for both, trophozoites and cysts ($p \leq 0.05$).<br>Conclusion: The voltages tested were effective for both cysts and trophozoites, given that the percentages of permeabilization were close, with no statistical significance between them, only with the control groups. Therefore, lower voltages may be tested to reach the same potential obtained, as a future possibility of an alternative treatment for AK.
**Order of Authors:**	Palloma Santiago Prates Pessoa, B.Sc.
	Raphael Barcelos
	Larissa Fagundes Pinto
	Denise de Freitas
	Mauro Campos
**Additional Information:**	
**Question**	**Response**
**Financial Disclosure**<br><br>Enter a financial disclosure statement that describes the sources of funding for the work included in this submission. Review	The author(s) received no specific funding for this work.

Powered by Editorial Manager® and ProduXion Manager® from Aries Systems Corporation

the submission guidelines for detailed requirements. View published research articles from *PLOS ONE* for specific examples.<br><br>This statement is required for submission and **will appear in the published article** if the submission is accepted. Please make sure it is accurate.<br><br>**Funded studies**<br>Enter a statement with the following details:<br>• Initials of the authors who received each award<br>• Grant numbers awarded to each author<br>• The full name of each funder<br>• URL of each funder website<br>• Did the sponsors or funders play any role in the study design, data collection and analysis, decision to publish, or preparation of the manuscript?<br><br>Did you receive funding for this work?	
**Competing Interests**<br><br>Use the instructions below to enter a competing interest statement for this submission. On behalf of all authors, disclose any competing interests that could be perceived to bias this work—acknowledging all financial support and any other relevant financial or non-financial competing interests.<br><br>This statement is required for submission and **will appear in the published article** if the submission is accepted. Please make sure it is accurate and that any funding sources listed in your Funding Information later in the submission form are also declared in your Financial Disclosure statement.<br><br>View published research articles from *PLOS ONE* for specific examples.	The authors have declared that no competing interests exist.

Powered by Editorial Manager® and ProduXion Manager® from Aries Systems Corporation

**NO authors have competing interests**<br><br>Enter: *The authors have declared that no competing interests exist.*<br><br>**Authors with competing interests**<br><br>Enter competing interest details beginning with this statement:<br><br>*I have read the journal's policy and the authors of this manuscript have the following competing interests: [insert competing interests here]*<br><br>* typeset	
**Ethics Statement**<br><br>Enter an ethics statement for this submission. This statement is required if the study involved:<br><br>• Human participants<br>• Human specimens or tissue<br>• Vertebrate animals or cephalopods<br>• Vertebrate embryos or tissues<br>• Field research<br><br>Write "N/A" if the submission does not require an ethics statement.<br><br>General guidance is provided below. Consult the submission guidelines for detailed instructions. **Make sure that all information entered here is included in the Methods section of the manuscript.**	N/A

Powered by Editorial Manager® and ProduXion Manager® from Aries Systems Corporation

**Format for specific study types**<br><br>**Human Subject Research (involving human participants and/or tissue)**<br>• Give the name of the institutional review board or ethics committee that approved the study<br>• Include the approval number and/or a statement indicating approval of this research<br>• Indicate the form of consent obtained (written/oral) or the reason that consent was not obtained (e.g. the data were analyzed anonymously)<br><br>**Animal Research (involving vertebrate animals, embryos or tissues)**<br>• Provide the name of the Institutional Animal Care and Use Committee (IACUC) or other relevant ethics board that reviewed the study protocol, and indicate whether they approved this research or granted a formal waiver of ethical approval<br>• Include an approval number if one was obtained<br>• If the study involved *non-human primates*, add *additional details* about animal welfare and steps taken to ameliorate suffering<br>• If anesthesia, euthanasia, or any kind of animal sacrifice is part of the study, include briefly which substances and/or methods were applied<br><br>**Field Research**<br><br>Include the following details if this study involves the collection of plant, animal, or other materials from a natural setting:<br>• Field permit number<br>• Name of the institution or relevant body that granted permission	
**Data Availability**<br><br>Authors are required to make all data underlying the findings described fully available, without restriction, and from the time of publication. PLOS allows rare exceptions to address legal and ethical concerns. See the PLOS Data Policy and FAQ for detailed information.	Yes - all data are fully available without restriction

Powered by Editorial Manager® and ProduXion Manager® from Aries Systems Corporation

A Data Availability Statement describing where the data can be found is required at submission. Your answers to this question constitute the Data Availability Statement and **will be published in the article**, if accepted.<br><br>**Important:** Stating 'data available on request from the author' is not sufficient. If your data are only available upon request, select 'No' for the first question and explain your exceptional situation in the text box.<br><br>Do the authors confirm that all data underlying the findings described in their manuscript are fully available without restriction?	
**Describe where the data may be found in full sentences. If you are copying our sample text, replace any instances of XXX with the appropriate details.**<br><br>• If the data are **held or will be held in a public repository**, include URLs, accession numbers or DOIs. If this information will only be available after acceptance, indicate this by ticking the box below. For example: *All XXX files are available from the XXX database (accession number(s) XXX, XXX.)*.<br>• If the data are all contained **within the manuscript and/or Supporting Information files**, enter the following: *All relevant data are within the manuscript and its Supporting Information files.*<br>• If neither of these applies but you are able to provide **details of access elsewhere**, with or without limitations, please do so. For example:<br><br>*Data cannot be shared publicly because of [XXX]. Data are available from the XXX Institutional Data Access / Ethics Committee (contact via XXX) for researchers who meet the criteria for access to confidential data.*<br><br>*The data underlying the results presented in the study are available from (include the name of the third party*	All relevant data are within the manuscript and its Supporting Information files.

Powered by Editorial Manager® and ProduXion Manager® from Aries Systems Corporation

and contact information or URL).<br>• This text is appropriate if the data are owned by a third party and authors do not have permission to share the data.<br><br>* typeset	
Additional data availability information:	

Powered by Editorial Manager® and ProduXion Manager® from Aries Systems Corporation

Manuscript Click here to access/download;Manuscript;Manuscript - palloma.docx

Effects of electroporation on *Acanthamoeba Polyphaga*

Eletroporation on *Acanthamoeba Polyphaga*

Palloma Santiago Prates Pessoa, Raphael Barcelos, Larissa Fagundes Pinto, Denise de Freitas, Mauro Campos

Department of Ophthalmology and Visual Sciences, Paulista School of Medicine, São Paulo Hospital, Federal University of São Paulo, São Paulo, SP, Brazil - UNIFESP

Corresponding author:

Mauro Campos, Department of Ophthalmology and Visual Sciences, Paulista School of Medicine, São Paulo Hospital, Federal University of São Paulo - UNIFESP, Rua Pedro de Toledo, 669 - Vila Clementino, São Paulo, SP, Brazil, Zip Code: 04039-032.

Phone number: +55 (11) 9-9215-2515

Email: mscampos@uol.com.br

Word-count: 3.561

Commercial Relationships Disclosures

P.S.P. Pessoa, None; **R. Barcelos,** None; **L.F. Pinto**, None; **D. Freitas**, None; **M. Campos,** None.

Abstract

Background: Species of the genus *Acanthamoeba* spp. are ubiquitous and can cause *Acanthamoeba* keratitis (AK), an opportunistic infection. Due to the growth in the number of AK cases, and therapies lasting an average of 6 months to an year, we are investigating the possibility of electrochemical treatment, which consists of destabilizing the membrane/wall of *Acanthamoeba* trophozoites and cysts to eliminate these forms more quickly. Therefore, we investigated whether electroporation could induce non-viable *Acanthamoeba* cysts and trophozoites in vitro.

Methods: Cysts and trophozoites of *Acanthamoeba polyphaga* (ATCC® 30461TM) were exposed in vitro to an electric field with intensities of 2000 volts and 2500 volts. The effect of electroporation on feasibility was assessed by counting in a Neubauer hemocytometer using Trypan blue 0,4% dye, and permeabilization was assessed by fluorescence microscopy using propidium iodide (PI) to identify permeabilized amoebas, associated with the nuclear dye 4',6-diamidino-2-phenylindole dihydrochloride (DAPI). The images were acquired on a Nikon Eclipse TI-U microscope using an excitation wavelength of 488/617 for PI and 340/488 nm for DAPI and analyzed using *ImageJ*.

Results: The results obtained demonstrated the loss of 40% of trophozoites electroporated at 2000V and 42% of those electroporated at 2500V, while for cysts the loss was respectively 13% and 16%. For permeabilization at 2000V, the rate was 55% for trophozoites and cysts ($p \leq 0.05$); while at 2500V it was 59% for both, trophozoites and cysts ($p \leq 0.05$).

Conclusion: The voltages tested were effective for both cysts and trophozoites, given that the percentages of permeabilization were close, with no statistical significance between them, only with the control groups. Therefore, lower voltages may be tested to reach the same potential obtained, as a future possibility of an alternative treatment for AK.

Introduction

Studies show that the genus *Acanthamoeba* spp. has amphizootic characteristics, i.e. they are free-living unicellular protozoa capable of living in various environments, such as water, soil, dust, air, hospital equipment, public and private places, where they occasionally invade hosts, causing disease [1-3].

According to the biological cycle, *Acanthamoeba* has two evolutionary forms: the trophozoite and the cyst. The trophozoites are metabolically active and infective, where they multiply, feed and invade host cells. Under extreme conditions of temperature, osmolarity, salinity, pH and lack of food, the trophozoites transform into cysts. These are latent and resistant forms, allowing them to remain and spread in the environment, as well as becoming chronic in cases of eye disease [1,3].

This protozoan can be classified according to the morphology of its cysts (Pussard and Pons classification, 1977) into three distinct groups including 25 species or based on 18S rDNA genetic sequencing, currently comprising 23 different genotypes (T1-T23), (4-8). Despite their almost universal presence throughout the environment, infections caused by *Acanthamoeba* in humans are relatively uncommon. But the combination of disruption of the corneal epithelium barrier, either through trauma or the use of contact lenses, and exposure to a sufficient inoculum of *Acanthamoeba* substantially increases the risk of keratitis (inflammation of the cornea). As a result, the patient may present with some initial non-specific symptoms, such as tearing, photophobia, blurred vision and intolerance to the use of contact lenses. There may also be blepharospasm, eyelid edema and corneal infiltrate, which can easily be confused with other ocular infections, expanding to severe cases with more specific symptoms, severe and painful ulcers in the corneal region, which can spread to the inner layers of the eyeball [5, 11-20].

The treatment of *Acanthamoeba* keratitis (AK) is prolonged, intensive and expensive, as it is a resistant etiologic agent. In order to eliminate trophozoites in the initial phase, as well as eradicating cysts and trophozoites throughout the therapeutic process, the approach during treatment involves the combination of two distinct classes of drugs: aromatic diamidines, which act on DNA synthesis (propamidine isethionate 0. 1% or hexamidine 0.1%), and cationic antiseptics that induce membrane lysis (biguanide 0.02% to 0.06% or chlorhexidine 0.02% to

0.2%). The most common combination is biguanide 0.02%, with or without propamidine 0.1%. Propamidine is only effective in the trophozoite phase, has no cysticidal properties and is only used in the first month of treatment. Biguanide, on the other hand, is often maintained for long periods of time, with the average duration of treatment varying from four to six months (it can extend from one to 26 months), showing certain efficacy against trophozoites and cysts. Sometimes, this type of therapeutic intervention is not able to eliminate the amoebas from the cornea and surgical interventions are necessary, thus demonstrating the urgency of searching for new methods or improvements in treatment, with the aim of making it less time-consuming for patients [5, 13, 14, 19, 21, 22, 26]. This led to the proposal of possibly incorporating electroceutical treatment in conjunction with the drugs already available.

The electroporation procedure consists of applying electric fields that temporarily alter the permeability and conductivity of the plasma membrane of biological cells. This process takes place inside the electroporator, where the intensity of the electric field, the number of short electrical pulses and the force applied can be configured to form hydrophilic pores, allowing the permeation of molecules that normally cannot cross the barrier imposed by the lipid bilayer. When only used to alternate the entry and exit of molecules, it is a reversible process, i.e. the membrane remains permeabilized for a few minutes and then closes, after which cellular equilibrium is re-established. With more exacerbated pulse parameters, the electroporation process becomes irreversible, as the cell is unable to restructure itself [27-31].

In recent years, these microorganisms have gained the attention of the scientific community due to the increase in the number of cases of eye infections. In this context, this study aims to analyze the opening of pores in the parasite wall in order to possibly facilitate the permeation of drugs through it in the future, which could become an effective treatment for cases of AK.

Methods

Description of the isolates of *Acanthamoeba polyphaga*

A standardized *Acanthamoeba* strain was used in this study: *Acanthamoeba Polyphaga* (*American Type Culture Collection*, ATCC® 30461™) obtained from a corneal scrape of a CA case in Texas, 1973.

Acanthamoeba Polyphaga Culture

The ATCC 30461 isolate was grown on non-nutrient agar medium supplemented with heat-inactivated *E. coli* (DH5α) at 25ºC. After these strains had been disentangled, the non-nutrient agar plate was washed with transport saline and the wash was centrifuged at 2000 RPM for 15 minutes. After centrifugation, the supernatant was discarded and the amoeba pellet re suspended in 1mL of culture medium, which was then inoculated into a 25 cm^2 culture flask containing 4mL of culture medium and incubated in an oven at 25ºC. To obtain the trophozoite forms, the isolate was grown in PYG (proteose-peptone - yeast extract - glucose) culture medium and, for the cystic forms, it was grown in Neff encystment medium.

Electroporation

Trophozoites and cysts of the ATCC 30461 isolate were counted in a Neubauer chamber and their concentration was adjusted to obtain an initial inoculum of $5x10^6$ trophozoites/cysts per mL of electroporation buffer (285mM sacarose, 0,7 $MgCl_2$, 1mM KlC, 10mM HEPES, 3Mm NaOH). Thus, 100µL of this inoculum ($5x10^5$ amoebas) were added to the electroporation cuvettes exposed to an electric field of 2000 and 2500V. Subsequently, methods for detecting electroporation involving fluorescence, described in detail below in item “Electroporation detection”, were carried out to standardize and evaluate the best voltage to be applied to *Acanthamoeba* cysts and trophozoites.

Electrochemical treatment

The *in vitro* experimental model was divided into the following groups: cysts and trophozoites controls (without electroporation); group 1: cysts and trophozoites electroporated at 2000 volts; group 2: cysts and trophozoites electroporated at 2500 volts.

Electroporation detection

To investigate the viability and permeabilization of electroporated cysts and trophozoites of isolate ATCC 30461, two tests were applied: feasibility and permeabilization, as described below.

Feasibility analysis

To assess the loss of cysts and trophozoites from the ATCC 30461 isolate resulting only from the shock applied, a separate analysis was first carried out counting the number of amoebas in each group before and after electroporation using Trypan blue 0.4% in a Neubauer hemocytometer. With the numbers obtained from the counts compared to the control groups, it was possible to determine separately the loss by electroporation from the loss of sample by the experimental process.

Permeabilization analysis

Subsequently, another analysis was carried out using the fluorescence intensity method. The electroporated cysts and trophozoites were plated in 96-well plates and stained with the fluorescent dye propidium iodide (PI; Sigma-Aldrich, USA).

The PI dye does not penetrate the wall/membrane of intact cysts and trophozoites, so if the amoebas turn red, it means that there has been a change in the permeability of these structures, allowing the dye to enter, and the greater the uptake of this fluorescence, consequently, the greater the damage to the wall or membrane of this isolate. The fluorescent dye 4',6-diamidino-2-phenylindol (DAPI; Sigma-Aldrich, USA), acts as a marker, coloring any cell with a blue nucleus. In summary, cysts and trophozoites electroporated in electroporation buffer + PI at a ratio of 1:100 (10µg/mL) were placed in 1.5ml microtubes in sterile PBS (1X) for 15 minutes at room temperature protected from light and then fixed with 4% paraformaldehyde (PFA; Synth, Brazil) for 15 minutes at 4°C protected from light, after which they were stained with DAPI dye at a ratio of 1:1000 for 10 minutes at room temperature. The 96-well plates were scanned for fluorescence intensity using an excitation wavelength of 488/617 for PI and 340/488 for DAPI and analysis was carried out using the IN Cell Analyzer 2200 device and counting using *Image J* software. The greater the intensity of the fluorescence captured, the greater the permeabilization. The images were acquired on a fluorescence microscope (Nikon Eclipse TI-U, Nikon, Tokyo, Japan) using the Nikon NIS-Elements platform, in which cysts and trophozoites stained in red (propidium iodide) were considered permeabilized. To reduce variation, all wells were run on the same plate at the same time, and the same batch of PI and DAPI was used for all experiments.

Statical Analysis

Statistical analyses were carried out using the GraphPad Prism 8.0 program. The two-way ANOVA test was used for multiple comparisons between groups. Values of $p < 0.05$ were considered statistically significant.

Results

Feasibility

From the initial inoculum of 200000 amoebas in 100µL there is a loss of 106083.3 in the controls due to centrifugation processes, from the remaining amount, discounting such loss values that differ from death electroporation, the calculations were carried out. The results obtained show that 40% of the trophozoites electroporated at 2000V and 42% of those electroporated at 2500V were lost, while for cysts the loss was 13% for 2000V and 16% for 2500V, as demonstrated in Figure 1.

Figure 1: Demonstration of viable and non-viable cysts and trophozoites in absolute numbers from the Neubauer chamber count.

Permeabilization

As for the permeabilization analysis, the results obtained from fluorescence analysis show that permeabilization at 2000V is 55% for trophozoites and 55% for cysts ($p \leq 0.05$); while at 2500V it is 59% for trophozoites and 59% for cysts ($p \leq 0.05$), as demonstrated in Figure 2, not being statistically significant between the electroporated groups, only when compared to the control group. Figures 3 and 4 show respectively the permaebilization of trophozoites and cysts.

Figure 2. Permeabilization of cysts and trophozoites of *A. Polyphaga* (ATCC 30461) electroporated at 2000 and 2500 volts. * indicates a significant difference between the control group and the groups electroporated at 2000 and 2500 volts ($P < 0.05$) (Two-way ANOVA).

Figure 3. *A. polyphaga* (ATCC 30461) trophozoites permeabilized with fluorescence dyes after electroporation. Fluorescence images acquired on a Nikon Eclipse TI-U microscope of *A.*

polyphaga trophozoites electroporated at 2000 and 2500 volts. DAPI: 4',6-diamidino-2-phenylindol, PI: propidium iodide (20x magnification).

Figure 4. *A. polyphaga* cysts (ATCC 30461) permeabilized with fluorescence dyes after electroporation. Fluorescence images acquired under a microscope of *A. polyphaga* trophozoites electroporated at 2000 and 2500 volts. DAPI: 4',6-diamidino-2-phenylindol, PI: propidium iodide (20x magnification).

Discussion

As far as we know, this is the first time that electroporation has been proposed in the field of ophthalmology as a method for the possible treatment of patients with *Acanthamoeba* keratitis. Despite the advances and discoveries about the disease, *Acanthamoeba* keratitis has remained significant over the years. Considering that avoiding risk factors and having an early diagnosis are still considered the most effective ways of combating the disease, and bearing in mind that from a more advanced stage of this pathology, treatment usually has a long duration and high toxicity, generating great discomfort for patients during this period, there is an urgent need to develop a shorter and more effective treatment [(5)]. This led to the idea of using electroporation to combat *Acanthamoeba*.

As is well known, *Acanthamoeba* is an amphizoic protozoan, widely distributed in the environment, with two evolutionary forms, the trophozoite and the cyst. The trophozoite represents the vegetative form of this protozoan, in which it reproduces by binary fission with movement and continuous changes in shape, in addition to producing human infection, which in cases of eye infection can occur from trauma and especially from the misuse of contact lenses that facilitate the invasion of the parasite, since the trophozoite uses its pseudopods to adhere to the surface of the contact lens. In unfavorable conditions, i.e. nutrient and oxygen deficiency, the trophozoite gradually becomes a cyst, with a very low rate of metabolism but with powerful resistance in the environment, its mobility decreases until it stops completely, the cytoplasm dehydrates and a dense double wall is formed, made up mainly of cellulose, which can withstand various types of conditions in the environment for several years while maintaining its pathogenicity. If conditions become favorable again, the cyst can transform back into a

trophozoite, leaving behind its double wall, and can thus reproduce again and cause infection. Therefore, being in an unsuitable environment, which can also be caused by the start of treatment, encystment will inevitably occur, which represents the biggest challenge in current treatments due to the difficulty the drugs have in penetrating the double layer of the cysts [1-5, 21, 22].

In order to improve the efficacy and shorten the treatment time of current therapeutic methods, electroceutical treatment has been proposed. The electroporation technique is a process that promotes the destabilization of the plasma membrane by means of electrical pulses. Depending on the intensity of the electrical pulses and the number of pulses applied, it is possible to make the disruption of the membrane irreversible, resulting in cell death, or reversible only with the formation of pores, allowing the exchange of external and internal substances through the membrane, making it possible for drugs to permeate more easily, which would enhance the treatment [27-31].

This study shows that the trophozoites left in ideal conditions of food temperature, multiplying considerably quickly, from the application of the electroporation method, using just a single pulse, made a significant 40% of the trophozoites electroporated at 2000V and 42% of those electroporated at 2500V unviable. Of the trophozoites that remained viable despite the shock, 55% of those electroporated at 2000V and 59% of those electroporated at 2500V were permeabilized.

With the protozoa that were left in a nutrient- and oxygen-deficient environment, the trophozoites gradually turned into cysts, and considering that this is the latent and resistance form, this study shows that the electroporation method using just a single pulse made 13% of the cysts electroporated at 2000V and 16% of those electroporated at 2500V unviable. Of the cysts that remained viable after the shock, 55% of those electroporated at 2000V and 59% of those electroporated at 2500V were permeabilized.

Thus, based on all the research carried out and the results obtained, it is possible to state that electroporation proved to be effective in combating cysts and trophozoites of *Acanthamoeba Polyphaga* at both voltages tested, considering the proximity of the percentages of permeabilization, with no statistical significance between them, only when compared with the control groups, justifying the relevance of this study in the current context. In the near future, lower

voltages and greater amounts of pulses will be tested in order to achieve the same potential already obtained or more, as a future alternative treatment possibility for *Acanthamoeba* keratitis.

Acknowledgments

We would like to express our gratitude to the CEPA, LOFT and INFAR research laboratories, as well as Professor Sang Won Han, for their valuable contributions to the development of the project.

References

1. Magliano AC, da Silva FM, Teixeira MM, Alfieri SC. Genotyping, physiological features and proteolytic activities of a potentially pathogenic *Acanthamoeba* sp. isolated from tap water in Brazil. Experimental Parasitology. 2009;123(3):231-5.
2. Trabelsi H, Dendana F, Sellami A, Sellami H, Cheikhrouhou F, Neji S, et al. Pathogenic free-living amoebae: epidemiology and clinical review. Pathol Biol (Paris). 2012;60(6):399-405.
3. Lorenzo-Morales J, Martín-Navarro CM, López-Arencibia A, Arnalich-Montiel F, Piñero JE, Valladares B. *Acanthamoeba* keratitis: an emerging disease gathering importance worldwide? Trends Parasitol. 2013;29(4):181-7.
4. Z. S, Wang. *Acanthamoeba* Keratitis: Diagnosis and Treatment. 1 ed. Beijing, China: Springer Nature Singapore; 2018 February 21. XV-116 p.
5. Fuerst PA, Booton GC, Crary M. Phylogenetic analysis and the evolution of the 18S rRNA gene typing system of *Acanthamoeba*. J Eukaryot Microbiol. 2015;62(1):69-84.
6. Corsaro D, Walochnik J, Köhsler M, Rott MB. *Acanthamoeba* misidentification and multiple labels: redefining genotypes T16, T19, and T20 and proposal for *Acanthamoeba micheli* sp. nov. (genotype T19). Parasitol Res. 2015;114(7):2481-90.
7. Hasni I, Andréani J, Colson P, La Scola B. Description of Virulent Factors and Horizontal Gene Transfers of Keratitis-Associated Amoeba Acanthamoeba Triangularis by Genome Analysis. Pathogens. 2020;9(3).

8. Booton GC, Visvesvara GS, Byers TJ, Kelly DJ, Fuerst PA. Identification and distribution of Acanthamoeba species genotypes associated with nonkeratitis infections. J Clin Microbiol. 2005;43(4):1689-93.
9. Putaporntip C, Kuamsab N, Nuprasert W, Rojrung R, Pattanawong U, Tia T, et al. Analysis of Acanthamoeba genotypes from public freshwater sources in Thailand reveals a new genotype, T23 Acanthamoeba bangkokensis sp. nov. Sci Rep. 2021;11(1):17290.
10. Garate M, Marchant J, Cubillos I, Cao Z, Khan NA, Panjwani N. In vitro pathogenicity of Acanthamoeba is associated with the expression of the mannose-binding protein. Invest Ophthalmol Vis Sci. 2006;47(3):1056-62.
11. Ibrahim YW, Boase DL, Cree IA. Factors affecting the epidemiology of Acanthamoeba keratitis. Ophthalmic Epidemiol. 2007;14(2):53-60.
12. Alvarenga LS, Freitas Dd, Hofling-Lima AL. Ceratite por Acanthamoeba. Arquivos brasileiros de oftalmologia. 2000;63(2):155-9.
13. Gomes ToS, Magnet A, Izquierdo F, Vaccaro L, Redondo F, Bueno S, et al. Acanthamoeba spp. in Contact Lenses from Healthy Individuals from Madrid, Spain. PLoS One. 2016;11(4):e0154246.
14. Kilvington S, Gray T, Dart J, Morlet N, Beeching JR, Frazer DG, et al. Acanthamoeba keratitis: the role of domestic tap water contamination in the United Kingdom. Invest Ophthalmol Vis Sci. 2004;45(1):165-9.
15. Ormerod LD, Smith RE. Contact lens-associated microbial keratitis. Arch Ophthalmol. 1986;104(1):79-83.
16. Thakur DV, Gaikwad UN. Microbial contamination of soft contact lenses & accessories in asymptomatic contact lens users. Indian J Med Res. 2014;140(2):307-9.
17. Lorenzo-Morales J, Khan NA, Walochnik J. An update on Acanthamoeba keratitis: diagnosis, pathogenesis and treatment. Parasite. 2015;22:10.
18. Maycock NJ, Jayaswal R. Update on Acanthamoeba Keratitis: Diagnosis, Treatment, and Outcomes. Cornea. 2016;35(5):713-20.
19. Panjwani N. Pathogenesis of Acanthamoeba keratitis. Ocul Surf. 2010;8(2):70-9.

20. Szentmáry N, Daas L, Shi L, Laurik KL, Lepper S, Milioti G, et al. Acanthamoeba keratitis - Clinical signs, differential diagnosis and treatment. J Curr Ophthalmol. 2019;31(1):16-23.
21. Carrijo-Carvalho LC, Sant'ana VP, Foronda AS, de Freitas D, de Souza Carvalho FR. Therapeutic agents and biocides for ocular infections by free-living amoebae of Acanthamoeba genus. Surv Ophthalmol. 2017;62(2):203-18.
22. Hau SC, Dart JK, Vesaluoma M, Parmar DN, Claerhout I, Bibi K, et al. Diagnostic accuracy of microbial keratitis with in vivo scanning laser confocal microscopy. Br J Ophthalmol. 2010;94(8):982-7.
23. Tu EY, Joslin CE, Sugar J, Booton GC, Shoff ME, Fuerst PA. The relative value of confocal microscopy and superficial corneal scrapings in the diagnosis of Acanthamoeba keratitis. Cornea. 2008;27(7):764-72.
24. Kheirkhah A, Satitpitakul V, Syed ZA, Müller R, Goyal S, Tu EY, et al. Factors Influencing the Diagnostic Accuracy of Laser-Scanning In Vivo Confocal Microscopy for Acanthamoeba Keratitis. Cornea. 2018;37(7):818-23.
25. Alkharashi M, Lindsley K, Law HA, Sikder S. Medical interventions for Acanthamoeba keratitis. Cochrane Database Syst Rev. 2015(2):CD010792.
26. Chen C, Smye SW, Robinson MP, Evans JA. Membrane electroporation theories: a review. Med Biol Eng Comput. 2006;44(1-2):5-14.
27. Escoffre JM, Portet T, Wasungu L, Teissié J, Dean D, Rols MP. What is (still not) known of the mechanism by which electroporation mediates gene transfer and expression in cells and tissues. Mol Biotechnol. 2009;41(3):286-95.
28. Kotnik T, Frey W, Sack M, Haberl Meglič S, Peterka M, Miklavčič D. Electroporation-based applications in biotechnology. Trends Biotechnol. 2015;33(8):480-8.
29. Kotnik T, Rems L, Tarek M, Miklavčič D. Membrane Electroporation and Electropermeabilization: Mechanisms and Models. Annu Rev Biophys. 2019;48:63-91.
30. Gehl J, Frandsen SK. [Electroporation-based therapies in the treatment of cancer]. Ugeskr Laeger. 2019;181(20A).
31. Lee GH, Lee JE, Park MK, Yu HS. Adhesion of Acanthamoeba on Silicone Hydrogel Contact Lenses. Cornea. 2016;35(5):663-8.

Click here to access/download;Figure;Figure1.tiff

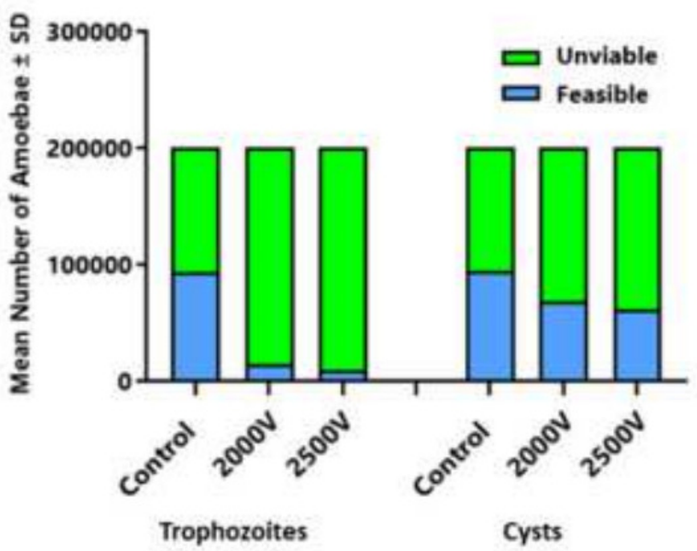

Figure2

Click here to access/download;Figure;Figure2.tiff

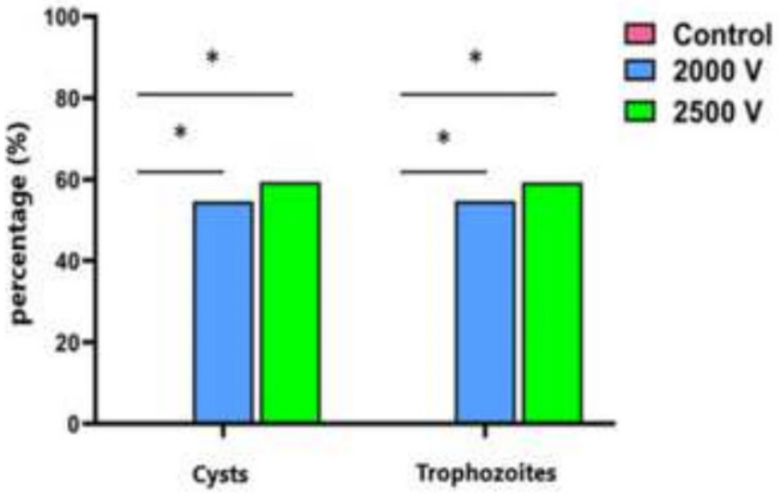

Figure3

Click here to access/download;Figure;Figure3.tiff

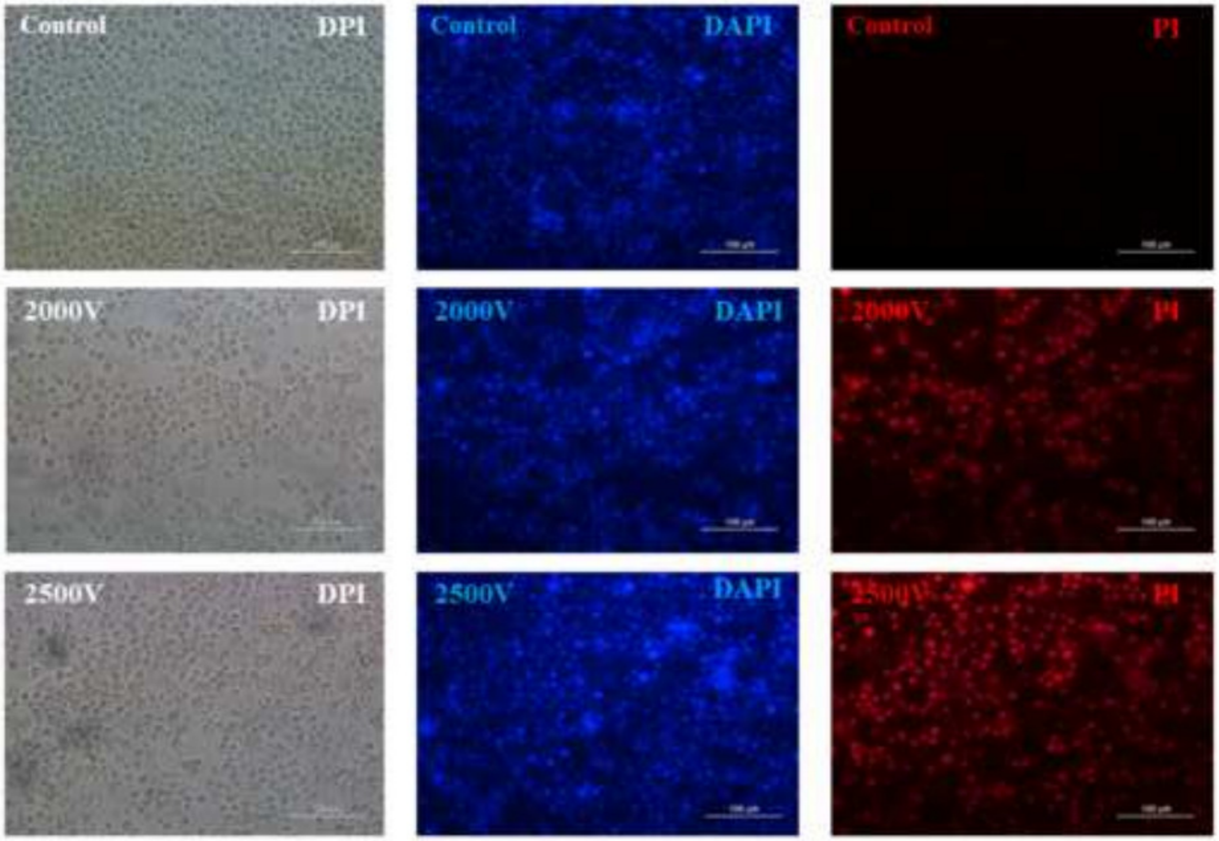

Figure4

Click here to access/download;Figure;Figure4.tiff

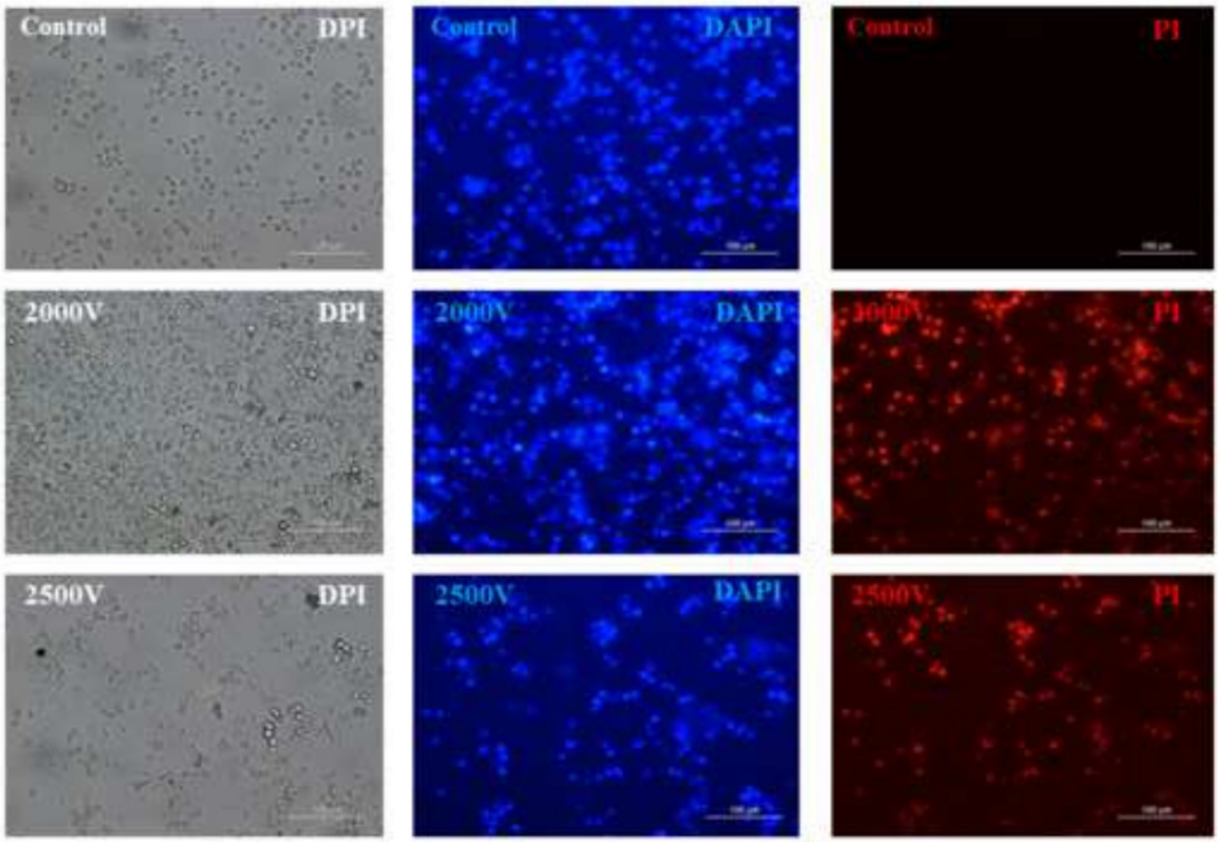

176780

ORIGINALITY REPORT

41%
SIMILARITY INDEX

PRIMARY SOURCES

1	www.medrxiv.org<br>Internet	477 words — 10%
2	sbpz.org.br<br>Internet	394 words — 9%
3	academics.su.edu.krd<br>Internet	241 words — 5%
4	www.research.ed.ac.uk<br>Internet	204 words — 4%
5	boris.unibe.ch<br>Internet	75 words — 2%
6	arpi.unipi.it<br>Internet	36 words — 1%
7	Majed Alkharashi, Kristina Lindsley, Hua Andrew Law, Shameema Sikder. "Medical interventions for acanthamoeba keratitis", Cochrane Database of Systematic Reviews, 2015<br>Crossref	31 words — 1%
8	link.springer.com<br>Internet	30 words — 1%
9	www.dovepress.com<br>Internet	

28 words — 1%

10 "Handbook of Electroporation", Springer Science and Business Media LLC, 2017
Crossref
26 words — 1%

11 Larissa F. Pinto, Marilise B. Rott, Mylena C. de Souza Barsch, Talita T. Rocchetti et al. " Adhesion of on Scleral Contact Lenses According to Lens Shape ", Investigative Ophthalmology & Visual Science, 2024
Crossref
26 words — 1%

12 www.spandidos-publications.com
Internet
24 words — 1%

13 immuno2023.sbi.org.br
Internet
21 words — < 1%

14 Allison Campolo, Paul Shannon, Monica Crary. "Evaluating Alternate Methods of Determining the Antimicrobial Efficacy of Contact Lens Care Products against Acanthamoeba Trophozoites", Pathogens, 2021
Crossref
18 words — < 1%

15 Naveed Ahmed Khan. "Acanthamoeba: biology and increasing importance in human health", FEMS Microbiology Reviews, 7/2006
Crossref
18 words — < 1%

16 Xuguang Sun. "Acanthamoeba Keratitis", Springer Science and Business Media LLC, 2018
Crossref
18 words — < 1%

17 www.frontiersin.org
Internet
18 words — < 1%

18 cms.galenos.com.tr

Internet

17 words — < 1%

19 lume.ufrgs.br
Internet
16 words — < 1%

20 repositorio.ufrn.br
Internet
16 words — < 1%

21 www.researchgate.net
Internet
16 words — < 1%

22 arro.anglia.ac.uk
Internet
12 words — < 1%

23 www.scienceopen.com
Internet
11 words — < 1%

24 Michel Drancourt, Luke Herbert. "Conjunctivitis, keratitis and infections of periorbital structures", Elsevier BV, 2010
Crossref
10 words — < 1%

25 arch.library.northwestern.edu
Internet
10 words — < 1%

26 e-sciencecentral.org
Internet
10 words — < 1%

27 molecularbrain.biomedcentral.com
Internet
10 words — < 1%

28 Sumeeta Khurana, Chayan Sharma. "Experimental models of Acanthamoeba keratitis", Indian Journal of Clinical and Experimental Ophthalmology, 2023
Crossref
9 words — < 1%

29	cev.org.br<br>Internet	9 words — < 1%
30	Anze Zupanic. "Numerical optimization of gene electrotransfer into muscle tissue", BioMedical Engineering OnLine, 2010<br>Crossref	8 words — < 1%
31	Cervero-Aragó, Sílvia, Sarah Rodríguez-Martínez, Antoni Puertas-Bennasar, and Rosa M. Araujo. "Effect of Common Drinking Water Disinfectants, Chlorine and Heat, on Free Legionella and Amoebae-Associated Legionella", PLoS ONE, 2015.<br>Crossref	8 words — < 1%
32	Sophie De Craene, Juliette Knoeri, Cristina Georgeon, Philippe Kestelyn, Vincent M. Borderie. "Assessment of Confocal Microscopy for the Diagnosis of Polymerase Chain Reaction–Positive Acanthamoeba Keratitis", Ophthalmology, 2018<br>Crossref	8 words — < 1%
33	Yukihiro Matsumoto, Asako Kodama, Eiki Goto, Tetsuya Kawakita, Murat Dogru, Kazuo Tsubota. "The Relation of Ocular Surface Irregularity and Visual Disturbance in Early Stage Acanthamoeba Keratitis", Eye & Contact Lens: Science & Clinical Practice, 2017<br>Crossref	8 words — < 1%
34	arquivosdeorl.org.br<br>Internet	8 words — < 1%
35	mdpi-res.com<br>Internet	8 words — < 1%
36	oaji.net<br>Internet	8 words — < 1%

37	Ágnes Füst, Jeannette Tóth, Gyula Simon, László Imre, Zoltán Z. Nagy. " Specificity of in Vivo Confocal Cornea Microscopy in Keratitis ", European Journal of Ophthalmology, 2016<br>Crossref	8 words — < 1%
38	Lyndon Jones, Nathan Efron, Kalika Bandamwar, Melissa Barnett et al. "TFOS Lifestyle: Impact of contact lenses on the ocular surface", The Ocular Surface, 2023<br>Crossref	7 words — < 1%
39	Muhammad Ilyas, Fiona Stapleton, Mark D. P. Willcox, Fiona Henriquez et al. "Epidemiology of and Genetic Factors Associated with Acanthamoeba Keratitis", Pathogens, 2024<br>Crossref	6 words — < 1%

EXCLUDE QUOTES	OFF	EXCLUDE SOURCES	OFF
EXCLUDE BIBLIOGRAPHY	ON	EXCLUDE MATCHES	OFF
