# Supplementary material for: Effects of electroporation on Acanthamoeba Polyphaga
Source: PLoS One. 2025 Feb 25;20(2):e0317409. doi: 10.1371/journal.pone.0317409 (PMC12072634; doi:10.1371/journal.pone.0317409)
Supplement: Attachment — Submitted filename: Response to Reviewers.doc [file pone.0317409.s002.doc]

São Paulo, July 12, 2024.

Dr Alireza Badirzadeh

PLOS ONE

Dear Dr. Badirzadeh:

Thank you very much for your response to the review of our manuscript, " Effects of electroporation on *Acanthamoeba*", submitted to PLOS ONE. We are very excited about the possibility of you accepting our paper!

We carefully read the excellent suggestions of each reviewer, agreed, incorporated, and justified them all.

Please find the attachments:

[1.] revised manuscript as a marked-up text (Word) file in which all changes have been tracked;

[2.] identical text as the clean text version;

[3.] a line-by-line response to all reviewers' remarks.

Once again, thank you so much for your attention to our manuscript and you can be sure that we did our BEST to get this acceptance!

Kind regards,

__________________________________

Dr. Mauro Campos

Professor of Ophthalmology and Visual Sciences, Federal University of São Paulo/School of Medicine (UNIFESP/EPM)

**Journal Requirements:**

1. When submitting your revision, we need you to address these additional requirements.

Please ensure that your manuscript meets PLOS ONE's style requirements, including those for file naming.

- After review, we believe that the manuscript fully complies with the PLOS ONE guidelines.

2. We note that your Data Availability Statement is currently as follows: All relevant data are within the manuscript and its Supporting Information files.

Please confirm at this time whether or not your submission contains all raw data required to replicate the results of your study. Authors must share the “minimal data set” for their submission.

- We confirm that all the minimum information necessary to replicate our study has been included in the manuscript. No data from the analysis has been omitted, and all experimental procedures are recorded in the laboratory minutes. All relevant data for analysis are available as defined by the minimum data set required for replication according to PLOS guidelines.

**Comments to the Author**

1. Is the manuscript technically sound, and do the data support the conclusions?

The manuscript must describe a technically sound piece of scientific research with data that supports the conclusions. Experiments must have been conducted rigorously, with appropriate controls, replication, and sample sizes. The conclusions must be drawn appropriately based on the data presented.

Reviewer #1: Partly

Reviewer #2: Yes

2. Has the statistical analysis been performed appropriately and rigorously?

Reviewer #1: I Don't Know

Reviewer #2: Yes

3. Have the authors made all data underlying the findings in their manuscript fully available?

The [PLOS Data policy](http://www.plosone.org/static/policies.action#sharing) requires authors to make all data underlying the findings described in their manuscript fully available without restriction, with rare exception (please refer to the Data Availability Statement in the manuscript PDF file). The data should be provided as part of the manuscript or its supporting information, or deposited to a public repository.

Reviewer #1: Yes

Reviewer #2: Yes

4. Is the manuscript presented in an intelligible fashion and written in standard English?

PLOS ONE does not copyedit accepted manuscripts, so the language in submitted articles must be clear, correct, and unambiguous. Any typographical or grammatical errors should be corrected at revision, so please note any specific errors here.

Reviewer #1: Yes

Reviewer #2: Yes

5. Review Comments to the Author

I confirm that the manuscript has been prepared with scientific rigor, and the data presented are supported by the proposed conclusions. We appreciate reviewer 2's comment on the adequacy and rigor of the statistical analysis carried out. We ensure that all methods have been implemented accurately and in accordance with established scientific standards. Thank you for the thorough review and for the time dedicated to evaluating our scientific work. I am at your disposal for any further clarifications that may arise.

**Reviewer #1:**

1. This article has previously been presented at the following congress: CAMPOS, M. S. Q. ; FREITAS, D. ; BARCELOS, R. ; PINTO, L. F. ; PESSOA, P. S. P. . EFFECTS OF ELECTROPORATION ON *Acanthamoeba Polyphaga*. 2023. (Apresentação de Trabalho/Congresso). Based on the link: <https://sbpz.org.br/wp-content/uploads/2023/09/livro-inteiro-04set.pdf>
   1.1. During the course of the study, it was partially presented at the XXXVI Annual Meeting of the Brazilian Society of Protozoology, 2023. This is the first full disclosure of the study.
2. There are some similarities in this article with other published articles.
   2.1. To the best of our knowledge, this is the first study designed for quantitative evaluation of the viability and permeabilization of *Acanthamoeba polyphaga* using the electroporation technique.

**Reviewer #2:**

**Abstract section**

1. Lack of information regarding the methods used for performing electroporation on cysts and trophozoites.
   1.1. The abstract has been revised to include detailed information on the method used to carry out electroporation on cysts and trophozoites, as requested.
2. Insufficient details about the presented results, including data interpretation and statistical distributions.
   2.1. The section on the results has been improved to provide a better interpretation of the data, including statistical distributions.
3. Need for technical enhancement in presenting the results in a manner that allows for more precise analysis and comprehensive interpretation.
   3.1. The presentation of the results has been modified to allow for more precise analysis and comprehensive interpretation.
4. Absence of sufficient information regarding the significance of the obtained results and the necessity for clearer articulation of this significance.
   4.1. It is believed that the summary now more clearly articulates the significance of the results obtained, explaining their relevance.
5. Requirement for delineating limitations and suggestions for future research in the abstract.
   5.1. The mention of future research has been removed. The conclusion now focuses on the interpretation that the research suggests the possibility of a complementary treatment to the standard treatment.

* In general, the abstract has been reformulated to meet all the requirements, respecting the established character limit.

**Introduction section**

1. Line 50: "genus *Acanthamoeba* spp." should be "genus *Acanthamoeba*" as "spp." indicates multiple species, which is not necessary here.
   1.1 "spp." has been removed
2. Line 54: Instead of "evolutionary forms," use "life cycle stages" for more accurate terminology.
   2.1 The modification has been made.
3. Line 57: Change "These are latent and resistant forms, allowing them to remain and spread in the environment, as well as becoming chronic in cases of eye disease" to "These are dormant and resistant forms, enabling them to persist and spread in the environment, and to cause chronic infections in cases of eye disease."

**3.1** The modification has been made.

4. Line 62: Replace "23 different genotypes (T1-T23)" with "23 distinct genotypes (T1-T23)."

**4.1** The modification has been made.

5. Line 64: "in humans are relatively uncommon" should be "are relatively uncommon in humans."
   5.1 The modification has been made.
6. Line 69: Change "which can easily be confused with other ocular infections" to "often confused with other ocular infections."

**6.1** The modification has been made.

7. Line 70: Replace "expanding to severe cases with more specific symptoms, severe and painful ulcers in the corneal region, which can spread to the inner layers of the eyeball" with "progressing to severe cases with more specific symptoms, including severe and painful corneal ulcers that can extend to the inner layers of the eyeball."

**7.1** The modification has been made.

8. Line 74: "throughout the therapeutic process" should be "during the treatment process."

**8.1** The modification has been made.

9. Line 85: "making it less time-consuming for patients" should be "reducing the treatment duration for patients."

**9.1** The modification has been made.

10. Line 88: "applying electric fields" should be "application of electric fields."

**10.1** The modification has been made.

11. Line 91: Change "configured to form hydrophilic pores" to "adjusted to form hydrophilic pores."

**11.1** The modification has been made.

12. Line 94: "and then closes, after which cellular equilibrium is re-established" should be "and then closes, allowing cellular equilibrium to be re-established."

**12.1** The modification has been made.

13. Line 95: Change "With more exacerbated pulse parameters, the electroporation process becomes irreversible, as the cell is unable to restructure itself" to "With higher pulse parameters, electroporation becomes irreversible, preventing the cell from restructuring".

**13.1** The modification has been made.

14. Ensure the reference to "Pussard and Pons classification, 1977" is accurate and from a reliable source.

**14.1** We apologize for the mistake; we have corrected it by adding the correct reference to the text.

“Putaporntip C, Kuamsab N, Nuprasert W, Rojrung R, Pattanawong U, Tia T, et al. Analysis of *Acanthamoeba* genotypes from public freshwater sources in Thailand reveals a new genotype, T23 *Acanthamoeba* bangkokensis sp. nov. Sci Rep. 2021;11(1):17290.”

15. Provide more details about the safety and efficacy of the electroporation method.

**15.1** As far as we know, this is the first time that electroporation has been proposed in the field of ophthalmology. Therefore, in the text, we address the safety and efficacy of the method based on consolidated research in other areas of medicine.

16. Mentioning the potential use of electroporation to facilitate treatment should be backed by supporting studies and scientific research.

**16.1** We draw on the successful application of electroporation in other areas of medicine to suggest a possible adjunctive treatment to the standard treatment for *Acanthamoeba* keratitis.

“Bonadies A, Bertozzi E, Cristiani R, Govoni F, Migliano E. Electrochemotherapy in Skin Malignancies of Head and Neck Cancer Patients: Clinical Efficacy and Aesthetic Benefits. Acta Dermato Venereologica. 2019;99(13):1246–52.”

“Zhang W, Wang W, Chai W, Luo X, Li J, Shi J, et al. Breast tissue ablation with irreversible electroporation in rabbits: A safety and feasibility study. Scarfi MR, editor. PLOS ONE [Internet]. 2017 Jul 21 [cited 2019 Dec 19];12(7):e0181555.”

“Jean-Pierre Tasu, Tougeron D, Marie-Pierre Rols. Irreversible electroporation and electrochemotherapy in oncology: State of the art. 2022 Nov 1;103(11):499–509.”

- The introduction is quite lengthy. Consider summarizing some sections and focusing on the most critical points to maintain reader engagement and clarity.
- After re-examining the introduction, we decided not to reduce it any further, as we believe that all the information it contains is of fundamental importance for the reader's understanding.

**Methodology Section:**

**-** The methodology section is generally well-organized, but there are some areas where clarity and detail can be improved. Additionally, consistent formatting and correct scientific terminology are essential.

1. Line 105: "A standardized Acanthamoeba strain was used in this study: Acanthamoeba Polyphaga (American Type Culture Collection, ATCC® 30461TM) obtained from a corneal scrape of a CA case in Texas, 1973."

- Change "CA case" to "keratitis case."
- Consider rephrasing for clarity: "A standardized strain of Acanthamoeba polyphaga (ATCC® 30461TM) was used in this study, obtained from a corneal scrape of a keratitis case in Texas, 1973."
  1.1 The modifications have been made.

2. Line 109: "The ATCC 30461 isolate was grown on non-nutrient agar medium supplemented with heat-inactivated E. coli (DH5α) at 25ºC."

- Remove the line break before "The ATCC 30461 isolate."
- "heat-inactivated" should be hyphenated.

**2.1** The modifications have been made.

3. Line 110: "After these strains had been disentangled, the non-nutrient agar plate was washed with transport saline and the wash was centrifuged at 2000 RPM for 15 minutes."

- "disentangled" is not the correct term. Use "harvested."
- Specify the exact centrifuge conditions: "washed with transport saline and centrifuged at 2000 RPM for 15 minutes."

**3.1** The modifications have been made.

4. Line 112: "After centrifugation, the supernatant was discarded and the amoeba pellet re suspended in 1mL of culture medium, which was then inoculated into a 25 cm2 culture flask containing 4mL of culture medium and incubated in an oven at 25ºC."

- "re suspended" should be "resuspended."
- Clarify "culture medium" to specify the type.

**4.1** The modification has been made.

5. Line 114: "To obtain the trophozoite forms, the isolate was grown in PYG (proteose-peptone - yeast extract - glucose) culture medium and, for the cystic forms, it was grown in Neff encystment medium."

- Clarify: "To obtain the trophozoite forms, the isolate was grown in PYG (proteose-peptone - yeast extract - glucose) culture medium. For cystic forms, it was grown in Neff's encystment medium."

**5.1** The modification has been made.

6. Line 119: "Trophozoites and cysts of the ATCC 30461 isolate were counted in a Neubauer chamber and their concentration was adjusted to obtain an initial inoculum of 5x106 trophozoites/cysts per mL of electroporation buffer (285mM sacarose, 0,7 MgCl2, 1mM KlC, 10mM HEPES, 3Mm NaOH)."

- Correct "sacarose" to "sucrose."
- Correct "0,7 MgCl2" to "0.7 mM MgCl2."
- Correct "KlC" to "KCl."
- Correct "3Mm NaOH" to "3 mM NaOH.""

**6.1** The modifications have been made.

7. Line 122: "Thus, 100μL of this inoculum (5x105 amoebas) were added to the electroporation cuvettes exposed to an electric field of 2000 and 2500V."

- Clarify: "Thus, 100μL of this inoculum (5x105 amoebas) was added to the electroporation cuvettes, which were then exposed to an electric field of 2000 or 2500V."

**7.1** The modification has been made.

8. Line 123: "Subsequently, methods for detecting electroporation involving fluorescence, described in detail below in item “Electroporation detection”, were carried out to standardize and evaluate the best voltage to be applied to Acanthamoeba cysts and trophozoites."

- Subsequently, fluorescence-based methods for detecting electroporation were carried out as described below in the “Electroporation detection” section, to standardize and evaluate the optimal voltage for Acanthamoeba cysts and trophozoites."

**8.1** The modification has been made.

9. Line 129: "The in vitro experimental model was divided into the following groups: cysts and trophozoites controls (without electroporation); group 1: cysts and trophozoites electroporated at 2000 volts; group 2: cysts and trophozoites electroporated at 2500 volts."

- Use bullet points or a clearer list format for readability:

a. Controls (cysts and trophozoites without electroporation)
b. Group 1: cysts and trophozoites electroporated at 2000 volts
c. Group 2: cysts and trophozoites electroporated at 2500 volts

**9.1** The modification has been made.

10. Line 138: "To assess the loss of cysts and trophozoites from the ATCC 30461 isolate resulting only from the shock applied, a separate analysis was first carried out counting the number of amoebas in each group before and after electroporation using Trypan blue 0.4% in a Neubauer hemocytometer."

- "resulting only from the shock applied" can be simplified: "To assess the loss of cysts and trophozoites due to the applied shock, a separate analysis was performed. Amoebas in each group were counted before and after electroporation using 0.4% Trypan blue in a Neubauer hemocytometer."

**10.1** The modification has been made.

11. Line 146: "Subsequently, another analysis was carried out using the fluorescence intensity method. The electroporated cysts and trophozoites were plated in 96-well plates and stained with the fluorescent dye propidium iodide (PI; Sigma-Aldrich, USA)."

- Simplify: "Another analysis was performed using fluorescence intensity. Electroporated cysts and trophozoites were plated in 96-well plates and stained with propidium iodide (PI; Sigma-Aldrich, USA)."

**11.1** The modification has been made.

12. Line 152: "The fluorescent dye 4',6-diamidino-2-phenylindol (DAPI; Sigma-Aldrich, USA), acts as a marker, coloring any cell with a blue nucleus."

- Correct spelling: "4',6-diamidino-2-phenylindole (DAPI; Sigma-Aldrich, USA), acts as a marker, staining the nucleus of any cell blue."

**12.1** The modification has been made.

13. Line 167: "Statistical analyses were carried out using the GraphPad Prism 8.0 program. The two-way ANOVA test was used for multiple comparisons between groups. Values of p < 0.05 were considered statistically significant."

- Minor correction: "Statistical analyses were carried out using GraphPad Prism 8.0. A two-way ANOVA test was used for multiple comparisons between groups. Values of p < 0.05 were considered statistically significant."

**13.1** The modification has been made.

**Discussion Section:**

1. Some concepts need to be explained in simpler terms to enhance reader comprehension.
   1.1 We have revised the section to explain some concepts in simpler terms in order to improve the reader's understanding. We have done our best to make the language more accessible without compromising technical precision.
2. Provide more specific numbers and detailed information about the experimental setup, methodology, and results. Additionally, ensure consistency in referencing previous studies.
   2.1 We provide more detailed information on the experimental setup, methodology and results. We ensure that the sources cited are clearly identified and correctly referenced.
3. Elaborate on certain aspects of the results and methods to provide a clearer understanding, especially regarding any discrepancies observed in the experiments.
   3.1 We have elaborated on aspects of the results and methods to provide a clearer understanding, especially in relation to any discrepancies observed in the experiments.
4. Offer a more detailed interpretation of the results, including comparisons with similar studies and a discussion of potential implications and limitations.
   4.1 We provide a more detailed interpretation of the results, including comparisons with studies that apply the technique in other areas of medicine, with the aim of providing a more in-depth and contextualized analysis.
5. Discuss the generalizability of the findings beyond the laboratory setting and their applicability in real-world clinical scenarios.
   5.1 We include in the discussion the importance of the results beyond the laboratory environment in addition to their possible applicability in practical clinical contexts in the real world and the necessary considerations for this application.

Overall, improvements in expressing concepts, more precise information about results, additional explanations, and a more logical interpretation of the results can enhance the paper.
As a reviewer of the manuscript, I recommend addressing the low quality of the images and graphs. It is essential that these visual elements be of higher quality to effectively convey the research findings. Therefore, I strongly advise the authors to improve the resolution and clarity of the images and graphs before finalizing the manuscript for publication. This enhancement will significantly enhance the overall presentation and comprehension of the study.

- Thank you for your detailed comments and suggestions. We have worked carefully to address each of the requests mentioned, providing additional explanations for a clear interpretation of this study.

In addition, we have taken the necessary steps to improve the quality of the images and graphs, ensuring that these visual elements present adequate clarity about the results of the research. We are confident that these improvements have contributed significantly to the presentation and understanding of the study.
